# Pulmonary Adenocarcinoma Mimicking Pneumonia in a Young Adult

**DOI:** 10.7759/cureus.35267

**Published:** 2023-02-21

**Authors:** Andrea C Marin, Ankita Prasad, Vraj Patel, Charles Lwoodsky, Sharon Hechter, Ayesha Imtiaz, Priya Patel, Viraj Shah, Jennifer Appiah, Pramil Cheriyath

**Affiliations:** 1 Internal Medicine, Hackensack Meridian Health Ocean Medical Center, Brick, USA; 2 Internal Medicine, Rajarshee Chhatrapati Shahu Maharaj Government Medical College, Kolhapur, IND

**Keywords:** lung cancer, pneumonia, histology, pneumonic adenocarcinoma, adenocarcinoma

## Abstract

Lung cancer is the third most common cancer in the United States. Lung adenocarcinoma is a subtype of non-small cell lung cancer. On computed tomography (CT) it can appear as ground glass nodules, consolidative opacity, or solid mass lesions located in the periphery. Because it can appear as a consolidation, it can sometimes be confused with an infectious process such as pneumonia. We present a case of a 27-year-old male initially diagnosed with pneumonia; however, three months later, when he presented to the hospital with worsening pleuritic chest pain, fever, and dyspnea after a bronchoscopy a week before admission, pathology was positive for adenocarcinoma.

## Introduction

Lung cancer is one of the most prevalent cancers worldwide. It is typically classified into small-cell lung cancer (SCLC) and the more prevalent non-small-cell lung cancer (NSCLC). Lung adenocarcinoma is a subtype of NSCLC that originates mainly in the mucosal glands and accounts for roughly 40% of all lung malignancies [1]. Still, when exposed to the appropriate set of driver mutations, most epithelial cells can be reprogrammed toward diverse lung cancer fates [2]. The average age of diagnosis for lung adenocarcinoma is 71 years, which is uncommon before age 20 [1]. Cigarette smoking, family history, and occupational exposure are known risk factors. These produce genetic alterations in the *p53* gene in up to 52% of instances resulting in the formation of NSCLC [3]. *EGFR*, *KRAS*, and *ALK* are additional typically linked gene alterations. A significant increase in lung adenocarcinoma in women has been attributed to smoking during the previous four decades [1].

Lung adenocarcinoma can appear as ground glass nodules, consolidative opacity, or solid mass lesions on computed tomography (CT) [4]. It is a glandular tumor with mucin-producing cells that stain positively for mucin on histology. Compared to those with SCLC, those with NSCLC have a poorer response to chemotherapy. This is the reason why patients with resectable tumors are treated surgically. Despite improved treatments, the five-year survival rate is less than 12% to 15% [1]. A late diagnosis might lead to tumor spread or an unresectable tumor, resulting in a poorer outcome.

## Case presentation

This patient is a 27-year-old male who presented to the hospital with fever, left-sided chest pain, productive cough, and dyspnea on exertion. He had been well till three months back when he started with a fever and cough followed by dyspnea on exertion and chest pain, and he has diagnosed with pneumonia. However, his clinical and lung findings failed to resolve completely even after completing the antibiotics, and he was diagnosed with unresolved pneumonia. He complained of night sweats for the last three months but had no weight loss or hemoptysis in the previous three months. He has received doxycycline for 21 days, azithromycin for five days, and levofloxacin for 14 days for supposed pneumonia but did not have much relief. He did not have any significant medical or surgical history. He was a non-smoker and non-alcoholic. There was no family history of malignancy. He denied any sick contacts or recent travel. He was a painter and worked with spray paint and denied using any mask for protection.

**Figure 1:**
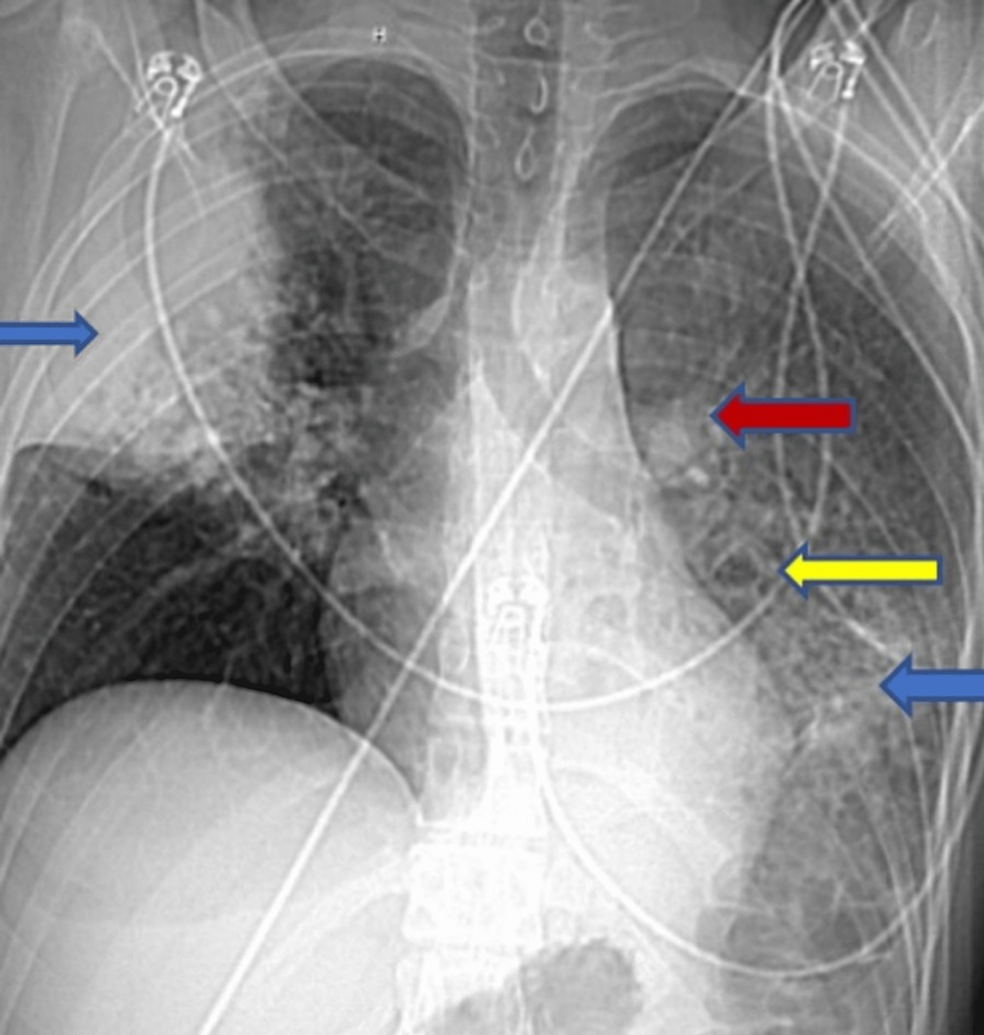
Chest X-ray image The image shows consolidation (blue arrow), nodularity (red arrow), hilar lymph node (yellow arrow).

**Figure 2:**
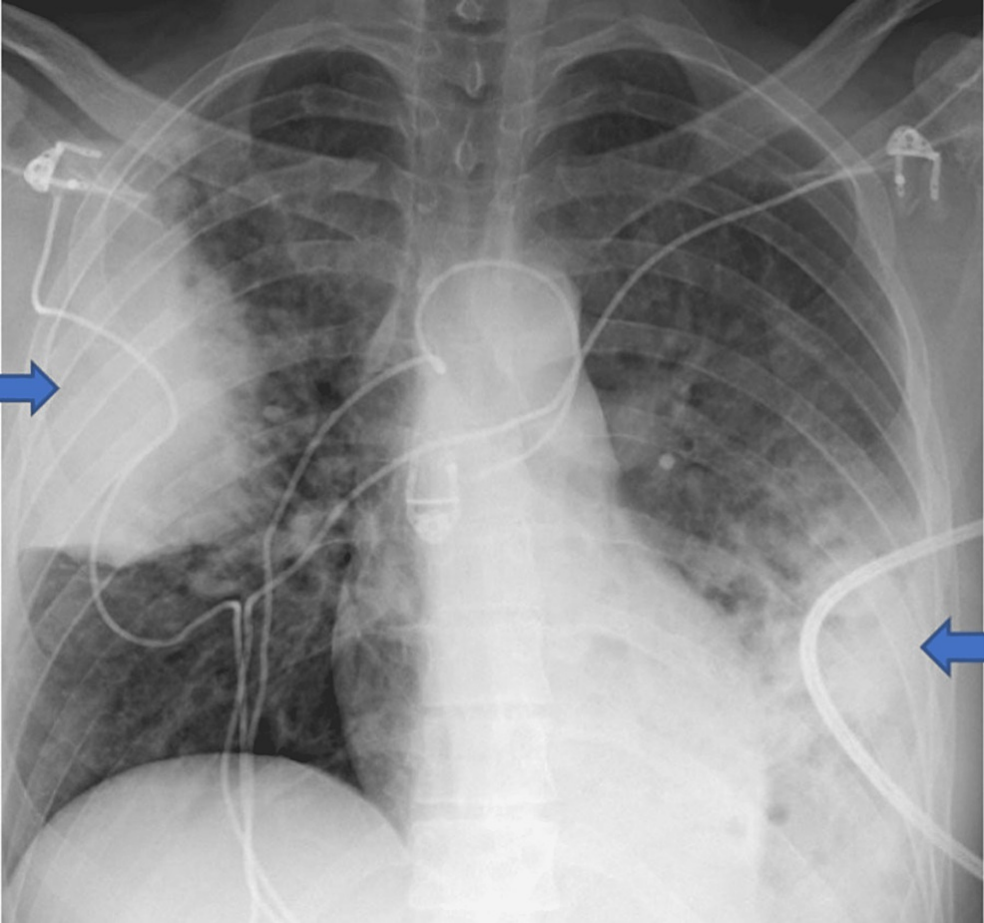
Chest X-ray showing consolidation Blue arrows show right and left lung consolidation.

He had stable vitals at admission. His heart rate was 84 beats per minute, his blood pressure was 118/76 mm Hg, his respiratory rate was 18 per minute, and his oxygen saturation at room air was 98 %. There was no pallor or any palpable lymph nodes; on auscultation, the chest had some scattered rhonchi; however, there was no distress or tracheal deviation, and other systemic examinations were within normal limits. His blood investigations ordered at the presentation showed a normal complete blood count and metabolic panels. His erythrocyte sedimentation rate (ESR) was 76 mm (<15mm/hr), C-reactive protein (CRP) was 6.5 mg/dl (<0.3 mg/dl), and Interferon-gamma release assay (IGRA) was negative for tuberculosis. His chest X-Ray showed persistent pulmonary consolidations in the left lower and middle lobes that were consistent with previous chest X-rays; however, the new one showed new nodularity (Figures 1, 2).

**Figure 3:**
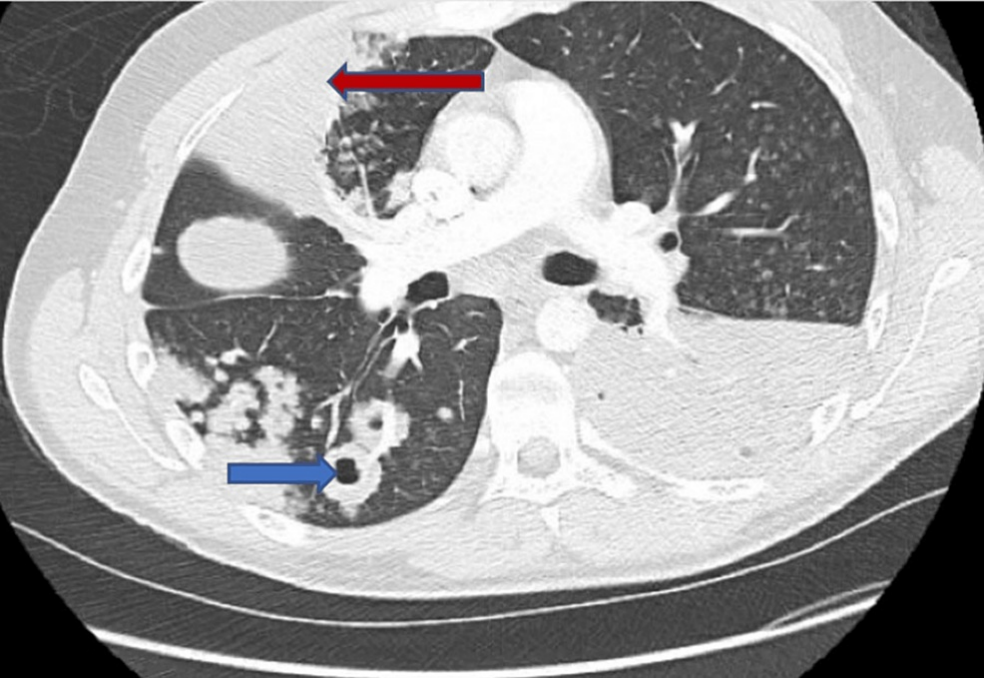
Computed tomography chest (axial images) The red arrow shows consolidation and the blue arrow shows evolving abscess.

**Figure 4:**
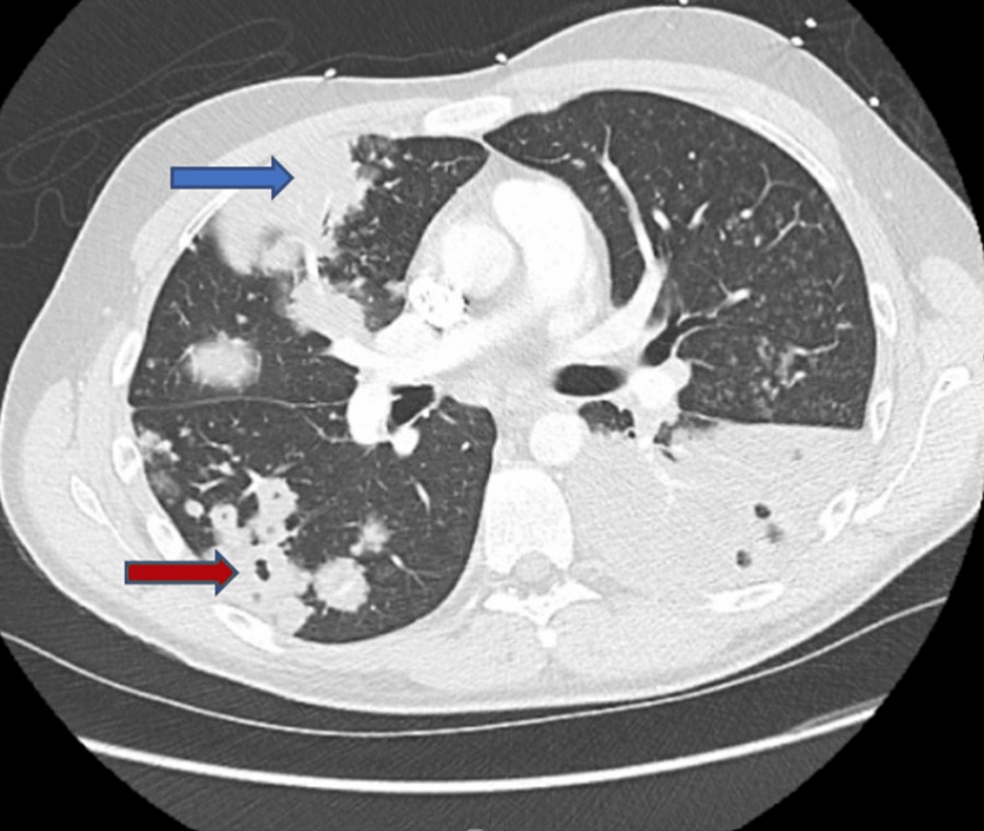
Computed tomography images of extensive multifocal pneumonia with dense right upper lobe and left lower lobe consolidation Multiple areas of breakdown and evolving abscesses within the consolidated left lower lobe (blue arrow). Numerous bilateral pulmonary nodules with cavitating nodules in the left lobe (red arrow).

Computed tomography of the chest showed extensive and dense multifocal pneumonic consolidation involving the right upper lobe and left lower lobe; abscesses were present within the consolidated left lower lobe with numerous bilateral pulmonary nodules and cavitating nodules in the right lower lobe (Figures 3, 4).

**Figure 5:**
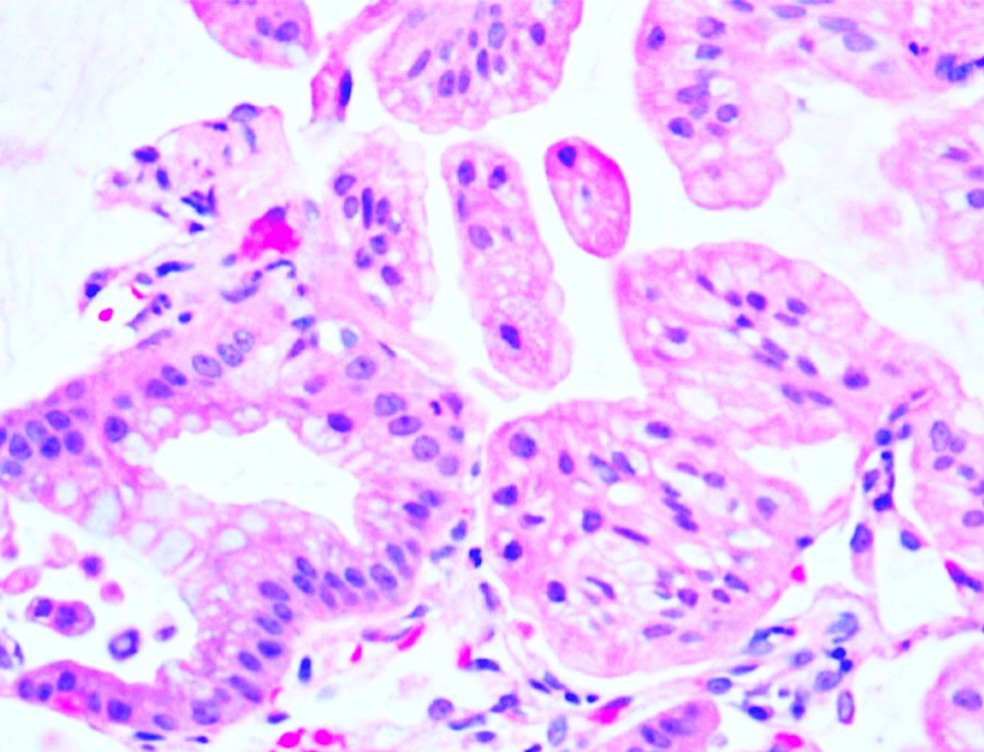
Histology (right upper lobe lung) Transbronchial biopsy reveals adenocarcinoma with mucinous features consistent with invasive mucinous carcinoma.

He underwent a bronchoscopy and transbronchial biopsy, and endobronchial ultrasound (EBUS) was performed at the level 7 lymph node due to his persistent symptoms. Bronchoscopy and EBUS were inconclusive; however, histopathology was first noted to be positive for adenocarcinoma (Figure 5).

**Figure 6:**
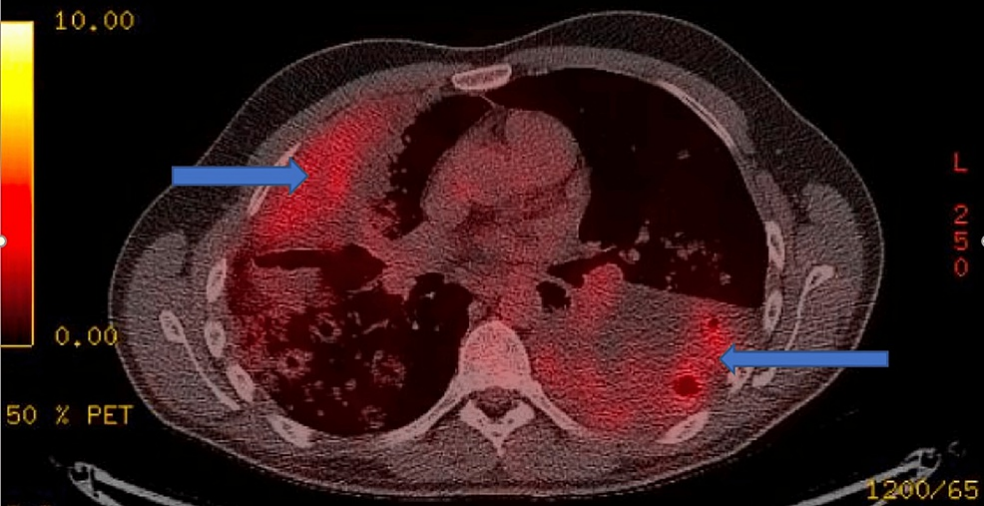
PET images PET: Positron emission tomography PET scan image shows increased tracer uptake (blue arrows).

A video-assisted thoracoscopic surgery (VATS) biopsy and a positron emission tomography (PET) scan were done for him (Figure 6).

An Infectious disease consultation was done at the presentation given the persistent fever. He was started on ampicillin and sulbactam, which was given for seven days after which he had a brief fever remission and the fever restarted within a week again. He was transferred to the care of an oncologist for further management follow up.

## Discussion

About 2% to 5% of all lung cancer cases are found in people under 40 years [5]. Amongst all lung cancers, 85% are non-small cell types, including adenocarcinomas, squamous cell carcinomas, and giant cell undifferentiated carcinomas [6]. Adenocarcinoma is the most prevalent type of lung cancer. Like all other lung cancers, it is related to tobacco use, but it is also the most frequently diagnosed lung cancer in nonsmokers, particularly women. It often grows more slowly than other lung tumors, although it can also spread in its early stages. Young adults are most frequently diagnosed with lung adenocarcinoma (age 25-40 years). Detterbeck et al. described pneumonic-type lung adenocarcinoma (P-ADC) as adenocarcinoma with pneumonia-like infiltration or consolidations involving regions in the lungs [7]. It is characterized by ground-glass opacity or consolidation on a chest CT [7] that resembles infectious or inflammatory lung disease. It can present with dyspnea, cough, and fever. Because of this, this type of lung adenocarcinoma is often misdiagnosed, especially since many people with this condition are nonsmokers. It is only after investigating the cause of non-resolving pneumonia that the diagnosis is made.

The imaging method of choice is a chest CT scan. CT images of peripheral consolidative pneumonia with surrounding nodules favor pneumonic-type adenocarcinoma instead of pneumonia. CT findings of an air-filled bronchus with stretching, squeezing, and enlargement of the branching angle or bulging interlobar fissures suggest pneumonic-type bronchioloalveolar carcinoma (BAC) rather than bacterial pneumonia [8]. It usually occurs in the lung periphery and, in many cases, may be found in scars or areas of chronic inflammation [1]. The final diagnosis is given by histopathological evaluation of the biopsy sample, which can be acquired by bronchoscopy or transthoracic surgery. Patients with no symptoms and a mass lesion on the chest radiograph had a better prognosis than those with symptoms and infiltrative signs on the chest radiograph [9].

Some studies have identified EGFR mutations in up to 75% of P-ADC, compared to 48.5% in patients with other forms of NSCLC [10]. The treatment of choice is a lobectomy or a pneumonectomy depending on the stage of the disease. Patients treated with lobectomy or bi-lobectomy fared better than those treated with pneumonectomy and chemotherapy. Total surgical resection in the absence of lymph node metastases was associated with a favorable outcome [9].

## Conclusions

P-ADC is often mistaken for infectious pneumonia because of its presentation. Early clinical manifestations of pneumonic lung cancer resemble most forms of pneumonia: cough, expectoration, shortness of breath, and the absence of particular symptoms. Imaging features include segmental, lobar, or pulmonary consolidation, similar to pneumonia. Cancer cells can grow and spread, which can cause secondary infections, bleeding in the lungs, pulmonary embolism, and other symptoms. Clinicians must remember this type of lung cancer, particularly in patients presenting with clinical and radiological signs indicative of unresolved pneumonia resistant to antimicrobial treatment.
